# Granulomatous splenic mass with necrosis revealing an EBV-positive inflammatory follicular dendritic cell sarcoma

**DOI:** 10.1093/jscr/rjac034

**Published:** 2022-05-05

**Authors:** Irena Antonia Ungureanu, Renato Micelli Lupinacci, Marie Parrens, Jean-François Emile

**Affiliations:** Department of Pathology, Ambroise-Paré Hospital, AP-HP, Boulogne-Billancourt 92100, France; EA4340-BECCOH Research Unit, Université de Versailles SQY, Versailles 78000, France; Department of Digestive and Oncologic Surgery, Ambroise-Paré Hospital, AP-HP, Boulogne-Billancourt 92100, France; Department of Pathology, CHU de Bordeaux, INSERM U1053, Université de Bordeaux, Bordeaux 33000, France; Department of Pathology, Ambroise-Paré Hospital, AP-HP, Boulogne-Billancourt 92100, France; EA4340-BECCOH Research Unit, Université de Versailles SQY, Versailles 78000, France

## Abstract

Epstein–Barr virus-positive inflammatory follicular dendritic cell sarcoma is a variant of follicular dendritic cell neoplasm most often arising in the liver or spleen. Two histological patterns can be identified in this variant, namely a granulomatous and an eosinophil-rich one. We present the case of a 69-year-old woman with a splenic mass. After being removed, the mass was gray-whitish with an area of necrosis. Histology showed a diffuse distribution of epithelioid granulomas in a background of a dense lymphoplasmacytic infiltrate. Rare atypical cells EBV+ and CD21+ were present in the intergranulomatous areas. Differential diagnosis for the granulomatous type EBV+ inflammatory follicular dendritic cell sarcoma includes infection, sarcoidosis, inflammatory myofibroblastic tumor, T cell lymphoma and vasculitis. The origin of this neoplasm is the follicular dendritic cell, and, due to its similarities with a myofibroblast, differential diagnosis can be challenging. Immunohistochemistry for dendritic markers and *in situ* hybridization for EBER remain diagnostic keys.

## INTRODUCTION

Epstein–Barr virus (EBV)-positive inflammatory follicular dendritic cell (FDC) sarcoma is a rare tumor of mesenchymal origin, occurring in young to middle-aged adults. Few cases are described in the literature, most of them arising in the liver or spleen [1–5]. Rare localizations such as colon [6, 7], peripancreatic region [2] or the pancreas [8] have also been reported.

Histologically, EBV+ inflammatory FDC sarcomas are characterized by neoplastic cells expressing dendritic markers in a background of a dense lymphoplasmacytic infiltrate. Rare variants may associate epithelioid granulomas or eosinophil-rich infiltrate which overshadow the neoplastic cells making the process of diagnosis extremely difficult [5]. There is a consistent positivity for EBV in neoplastic cells [2, 9].

We present a rare granulomatous variant of a splenic EBV+ inflammatory FDC sarcoma.

## CASE REPORT

**Figure 1:**
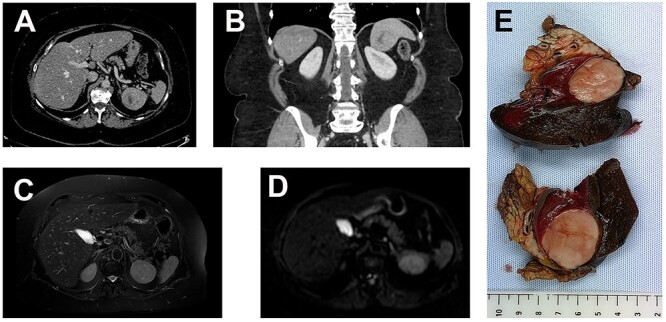
(**A**, axial) and (**B**, coronal), portal venous phase CT-scan showing a 43×40×39 mm lesion of the inferior pole of the spleen. The lesion is heterogeneous with a center of necrotic appearance, without calcification and enhancement after injection of the contrast product. A well-defined splenic lesion with high signal intensity on T2-weighted (**C**) and diffusion-weighted (**D**) MRI. (**E**) The macroscopic appearance: tumor is solid, yellow-tan on cut surface with well-circumscribed borders.

A 69-year-old woman presented to the hospital in the context of surveillance for sigmoidian diverticulitis. A Computed Tomography (CT) scan was performed and a splenic mass was found incidentally. It was absent on the previous CT scan performed 12 months earlier. The mass was solid and it was located at the splenopancreatic border (Fig. 1), without specific characteristics on Magnetic Resonance Imaging (MRI) and not hypermetabolic on PETscan. The patient had a history of a treated breast cancer, so the first hypothesis was that of a metastasis of breast carcinoma. A biopsy was performed which showed necrosis and massive inflammation consisting of plasma cells and lymphocytes, with no epithelial cells. The patient was in a good general state. Blood tests related to a possible infection were negative. The C-reactive protein was elevated (8 mg/L). The final decision was to perform a splenectomy and caudal pancreatectomy.

### Pathologic Findings

Grossly, the dimensions of resected spleen were in the right range. The tumor measured 6 cm in diameter; it was gray-whitish with areas of necrosis (Fig. 1). It was encapsulated and localized inside the splenic parenchyma with ‘pushing margin’ effect toward the pancreas without invading it.

**Figure 2:**
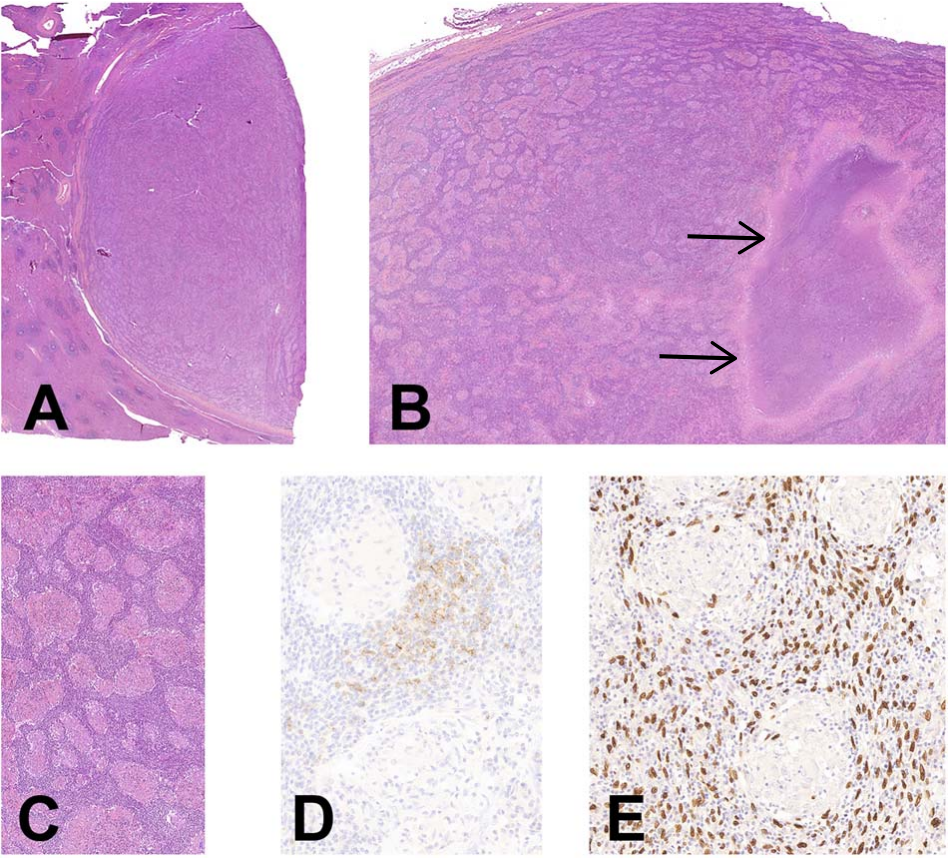
(**A**) Histology of EBV+ inflammatory FDC sarcoma. Low magnification showing the well delimitation of the tumor (at right) from the splenic parenchyma (at left), *H&E,* ×7. (**B**) The granulomatous appearance is well-noticed with a large area of necrosis (arrows), *H&E,* ×14. (**C**) There is an abundant lymphoplasmacytic infiltrate between the noncaseating epithelioid granulomas, *H&E,* ×90. (**D**) CD21 highlights the neoplastic FDCs situated in the intergranulomatous areas, *CD21 immunohistochemistry,* ×200. (**E**) Corresponding image of **D** showing an intense positivity of neoplastic cells for EBV, *EBER hybridization,* ×*200* (**E**).

Histologically, the entire tumor was represented by numerous noncaseating epithelioid granulomas with multinucleated giant cells in a background of a dense lymphoplasmacytic infiltrate. A large area of necrosis was associated. Between granulomas, rare atypical cells with large oval-shaped and vesicular chromatin nuclei were hardly noticed, being obscured by the dense inflammation (Fig. 2). Some vessels exhibited a hyalinization of the wall with intravascular thrombi, without vasculitis. Few scattered eosinophils were present.

Periodic acid-Schiff stain (PAS) and Ziehl stains did not reveal any germ. Immunohistochemistry for Anaplastic Lymphoma Kinase (ALK) and Human herpesvirus 8 (HHV8) was negative. The *in situ* hybridization for EBV was strongly positive in the atypical cells situated in the inter granulomatous areas, which highlighted large atypical and oval-shaped nuclei. Some of these cells were expressing CD21 and were slightly positive for the smooth muscle actine (SMA) (Fig. 2). CD35 was negative. Plasma cells had a polytypic expression of light chains, and the maximal density of IgG4 was 58/HPF. No *BRAF* mutation nor T cell clonality were detected.

## DISCUSSION

FDC sarcoma can be easily misdiagnosed. It is generally characterized by a slow growing mass. However, in our case, the absence of a splenic mass 1 year before suggests a quite fast development of this tumor. Granulomatous type is rare, being represented by only 9% of EBV+ inflammatory FDC sarcomas [2, 5]. Differential diagnosis for the granulomatous type includes: infection (mycobacterial or fungal), sarcoidosis, inflammatory myofibroblastic tumor (IMT, previously also known as inflammatory pseudotumor), T cell lymphoma and vasculitis.

EBV+ inflammatory FDC sarcoma is a neoplastic proliferation of FDCs, so the distinguishing element between this and other conditions is the atypical spindle/ovoid cells expressing some dendritic markers and lacking expression of other cell type lineage. However, it is difficult to spot the atypical spindle cells in an inflammatory background, because reactive myofibroblasts may mimic the morphology of a FDC. Detection of blood vessels with fibrinoid deposits can also be a diagnostic hint in favor of this tumor [5], but they were rare in our case.

Immunohistochemistry for dendritic markers is recommended: conventional markers such as CD21, CD23, CD35 and newly described markers such as PD-40, clusterin [4, 10]. Sometimes dendritic markers show a weak staining, which can hinder the differential diagnosis between EBV+ inflammatory FDC sarcoma and IMT. This can be explained by an overlapping immunophenotype between myofibroblastic cells and FDCs. The expression of SMA in an EBV+ inflammatory FDC sarcoma is thought to be due to a switching of markers between fibroblastic reticular cells and FDCs [4]. In our case, the neoplastic cells showed a weak expression of SMA and CD21 and a negativity for CD35.

Positivity for EBV is a major diagnostic key for EBV+ inflammatory FDC sarcomas and was observed in almost all reported cases. By contrast, EBV positivity is extremely rare in IMT. IMT is defined as a neoplastic proliferation of spindle cells associated with frequent *ALK* translocation [11]. EBV expression has also been reported in some leiomyosarcomas occurring in immunocompromised [12, 13]; however, these tumors have fasciculate architecture and cells have an eosinophilic cytoplasm.

Molecular characteristics have been described. The existence of a *BRAF* mutation in these tumors may help to differentiate between FDC sarcoma and inflammatory pseudotumor. Indeed, Go *et al*. reported BRAFV600E mutations in 2/5 EBV+ inflammatory FDC sarcomas, contrasting with 0/8 in IMT [14].

Both conventional type and the EBV+ inflammatory FDC sarcoma have an indolent behavior, surgery being a curative treatment. However, in intraabdominal localizations, classical FDC sarcoma appears to have a 40–50% rate of recurrence [15]. In a published series of six cases by Ji-Young Choe *et al.*, all patients with EBV+ inflammatory FDC sarcoma were alive with no evidence of disease during 8–78 months after the surgical treatment [4]. Another series of cases describes an 18.5% recurrence rate and one death [10]. Our patient is alive without signs of disease 5 months after the treatment.

EBV+ inflammatory FDC sarcoma is an unusual tumor, and rare variants rich in epithelioid granulomas should be considered when facing a granulomatous splenic tumor. The presence of an inflammatory granulomatous background makes the process of diagnosis extremely difficult. Immunohistochemistry for dendritic markers and *in situ* hybridization for EBER (EBV Encoded Ribonucleic acid) remain diagnostic keys.

## CONFLICT OF INTEREST STATEMENT

None declared.
